# Cardiovascular risk factors among retired attendees visiting primary care clinics

**DOI:** 10.12669/pjms.303.4269

**Published:** 2014

**Authors:** Yousef Abdullah Al Turki

**Affiliations:** 1 Dr. Yousef Abdullah al Turki, MBBS,DPHC, ABFM, Associate Professor and Consultant Family Medicine,Department of Family and Community Medicine,College of Medicine, King Saud University, P.O Box 28054 Riyadh 11437 Saudi Arabia.

**Keywords:** Cardiovascular risk factors, Retired attendees, Primary care

## Abstract

***Objective:*** The aim of this study was to highlight cardiovascular risk factors among retired attendees attending a primary care clinic, Riyadh, Saudi Arabia.

***Methods:***A cross sectional study was conducted from Januaryto February 2013 at Primary Care Clinics of King Khalid University Hospital and College of Medicine, King Saud University, Riyadh, Saudi Arabia. All retired attendees were interviewed by family physician, and their duration of retirement was determined. Their cardiovascular risk factors were confirmed from their medical records. The cardiovascular risk factors included history of diabetes mellitus, hypertension, dyslipidemia, obesity, and smoking. Their weight and height were recorded during the consultation and Body Mass Index was calculated to decide about those classified as obesity ≥ 30 All data were entered and analyzed using statistical package of social science SPSS version 17 software.

***Results: ***The present study showed that 19.5% of retired attendees presenting at primary care clinic were early retired before the age of 60 years, while 80.5% were normally retired. The prevalence of cardiovascular risk factors showed: Hypertension among 73% attendees, Diabetes Mellitus in 67%, dyslipidemia in 71%, Obesity 29%, and Smoking 13% of the patients.

***Conclusion:***This study concluded that cardiovascular risk factors among retired attendees of a primary care clinic are common, and need to be taken in to priority consideration while improving the health care of retired people.

## INTRODUCTION

Retirement is a period of life transition involving developmental and social psychological change in identity and preferences among retired people. However, there is little evidence and research data about the impact of the retirement transition on health outcomes.^1^^,^^2^ Also it is worthwhile to mention that increased life expectancy combined with a decline in average retirement age increased the proportion of an individual's life spent in retirement. This social change imposes many challenges for the financial sustainability of social systems. Moreover, this extended retirement period raises questions about its potential consequences on the physical, social and psychological health of the retired people, which may in turn affect long-term care expenditures.^3^^-^^11^However, empirical evidence on retirement effects on health remains inconsistent. Some studies have suggested a beneficial effect, at least in some groups, others an adverse effect, and still others no effect of retirement on health. This lack of consistent evidence is problematic given the drive to raise the retirement age in most developed countries.^12^^-^^16^ Some local studies in Saudi Arabia emphasized about the importance of studying cardiovascular risk factors in Saudi population and especially in old age group ^17^^-^^19^

The studies of health status of retired people in Saudi Arabia was limited and need to be study in depth especially considering cardiovascular risk factors assessment like diabetes mellitus, hypertension, dyslipidemia, obesity, and smoking, which need to be explored and highlighted among retired people in the community**.** The result of this study will might help the Ministry of health and other health related sector to plan to decrease the burden of cardiovascular risk factorsinretired patients and their families.^20^^-^^23^ The aim of this study was to assess the frequency of cardiovascular risk factors among retired attendees visiting primary care clinic, Riyadh, Saudi Arabia.

## METHODS

This was a cross sectional study was conducted during January and February 2013 at a Primary Care Clinics of King Khalid University Hospital and College of Medicine, King Saud University, Riyadh, Saudi Arabia.The study protocol was reviewed and approved by the Institutional Review Boardof King Saud University College of Medicine ,and approved by the Ethical Committee, Research project Number E-12-850.Informed consent for participation in the study was obtained from all participants. All retired attendees visiting a primary care clinic were interviewed by family physician, and their duration of retirement was determined. Two hundred participants were involved in the study, the data collection was through interviewing the participants, reviewing their medical records and by answering questionnaire about their socio demographic and retirement history, Their cardiovascular risk factors were confirmed from their medical records. The cardiovascular risk factors included history of diabetes mellitus, hypertension, dyslipidemia, obesity, and smoking. Their weight and height were recorded during the consultation and Body Mass Index was calculated (according to the World health organization WHO classification). All data were entered and analyzed using statistical package of social science SPSS version 17 software. 

## RESULTS

The present study showed that 19.5% of retired attendees to a primary care clinic were early retired before the age of 60 years, while 80.5% were normally retired. The study showed that 35% of them were retired for less than 5 years, while 50% of them were retired for 5-15 years, and only 15% were retired for more than 15 years. The study showed that 76% of them were married, 18.5% were widow, 4.5% were divorced, and only 1% were single. The majority of participants 96% were living with their families, and only 4% were living lone. The prevalence ofcardiovascular risk factors among retired attendees attending primary care clinic showed: Hypertension 73% as most common risk factor followed by dyslipidemia in 71% attendees and diabetes mellitus among 67% patients, obesity was present in 29% and smoking in 13%.When inquiring the satisfaction level a total of52.5% of retired attendees were satisfied and happy about their retirement, while 39% of retired attendees were unsatisfied and unhappy about their retirement status, and 8.5% were partially satisfied. All detailed findings of the study are shown in Tables I-III.

**Table-I:** Age distribution of retired attendees

*Age groups (years)*	*Frequency*	*Percentage %*
< 60	39	19.5
60 – 69	100	50
70 – 80	49	24.5
>80	12	6
Total	200	100

**Table-II:** Cardiovascular risk factors among retired participants

*Cardiovascular risk factors*	*Frequency*	*Percentage %*
Diabetes Mellitus	134	67
Hypertension	146	73
Dyslipidemia	142	71
Obesity	58	29
Smoking	26	13

**Table-III:** Cross tabulation between cardiovascular risk factors and different variables (P value

*Variables*	*Diabetes Mellitus*	*Hypertension*	*Dyslipidemia*	*Obesity*	*Smoking*
Age groups	0.252	0.007	0.139	0.025	0.025
Duration of retirement	0.098	0.002	0.296	0.489	0.071
Marital status	0.847	0.049	0.867	0.377	0.055
Retirement satisfaction	0.087	0.804	0.063	0.835	0.0001
Living status	0.782	0.897	0.294	0.294	0.966
Retirement status	0.142	0.003	0.025	0.002	0.009

P value is consider significant if it is less than 0.05%.

## DISCUSSION

The present study showed that 19.5% of retired attendees presenting to primary care clinic were early retired before the age of 60 years, while 80.5% were normally retired.Astudy showed that those who perceived their retirement to be voluntary had higher life satisfaction scores and rated themselves as healthier (both physically and mentally) than those who perceived their retirement as involuntary.^24^ In this study 52.5% of retired attendees were satisfied and happy about their retirement, while 39% of retired attendees were unsatisfied and unhappy about their retirement status, and 8.5% were partially satisfied.

Even in this study when cross tabulation between retirement status either early or normal and their satisfaction about retirement, the p value was 0.806 which is statistically not significant. While the retirement status was significantly associated with cardiovascular risk factors as p value were less than 0.05% in hypertension, dyslipidemia, obesity, and smoking.

This study also show on assessment of cardiovascular risk factors among retired attendees visiting primary care clinics the prevalence of hypertension was found to be most common followed by Dyslipidemia 71% and Diabetes Mellitus 67%, while 29% were obese and 13% smokers. These results emphasize the increasing frequency of these diseases. However, this may be related to increased age as most of attendees presenting are above age 60.Another factor leading to increased frequency might be dissatisfaction and social stress or financial difficulty. One multi center case control study conducted in 52 countries from 262 centers in Asia, Europe, the Middle East, Africa, Australia, and North and South America which conclude that presence of psychosocial stressors is associated with increased cardiovascular risk, suggesting that approaches aimed at modifying psychosocial stressors should be developed to decrease cardiovascular risk.^25^

About 96% of participants were living with their families, while only 4% were living a lone, which is an important aspect especially for retired people as studies have showed that the family is the major social support of the old person and emphasize the importance of family support in social life.^26^^-^^28^ In this study only 52.5% of retired attendees were satisfied about their retirement, so those who were unsatisfied might have some stress related to social life or financial reasons, or related to their illness. Also cross tabulation between retirement satisfactions and cardiovascular risk factors showed that P value were not statistically significant except with smoking(0.001), and this might explain the prevalence of 13% smokers among retired attendees as some of them tried to relieve stress by continue to smoke.This might not be related to retirement period as different studies have showed that smoking was common at different age groupsin Saudi Arabia.^29^^-^^32^ The prevalence of diabetes mellitus, hypertension, dyslipidemia, and obesity among retired attendees to a primary care clinic were high in this study which need to be taken into consideration in health planning to improve the health status of people in Saudi Arabia during retirement. Another study have showed that there is a high prevalence of diabetes mellitus, obesity, hypertension, and smoking in the Middle East.^33^

Cardiovascular disease is responsible for approximately one-third of deaths worldwide, and this figure will increase in both developing and developed countries as risk factors for the disease primarily dyslipidemia, hypertension, obesity, diabetes, physical inactivity, poor diet, and smoking continue to increase.^34^ In this study the prevalence of obesity (BMI≥30)among retired attendees were 29% which could be due to decrease in activities during retirement age and because of sedentary life styles. In Saudi Arabia there is lack of health research concerning retirement period which need to be considered in future exploration of different health issues among retired people.

## CONCLUSION

Cardiovascular risk factors among retired attendees of a primary care clinic might be common, which need to be taken in to priority consideration while improving the health care of retired people. Further large community based study is recommended.

## Notes

***Limitation of the study:*** One of the limitation of this study is that it is done in only one center, and further research through multi centers and community based study can explore in depth cardiovascular risk factors among retired people in the community.

## Notes

***Funding:*** None.
